# Tristetraprolin disables prostate cancer maintenance by impairing proliferation and metabolic function

**DOI:** 10.18632/oncotarget.13128

**Published:** 2016-11-05

**Authors:** Anders E. Berglund, Kristen E.N. Scott, Weimin Li, Chunying Yang, Mario R. Fernandez, Franz X. Schaub, John L. Cleveland, Robert J. Rounbehler

**Affiliations:** ^1^ Department of Biostatistics and Bioinformatics, H. Lee Moffitt Cancer Center & Research Institute, Tampa, Florida, USA; ^2^ Department of Tumor Biology, H. Lee Moffitt Cancer Center & Research Institute, Tampa, Florida, USA; ^3^ Department of Oncologic Sciences, University of South Florida, Tampa, Florida, USA

**Keywords:** prostate cancer, tristetraprolin, TTP, metabolism, proliferation

## Abstract

Tristetraprolin (TTP) is an RNA-binding protein that post-transcriptionally suppresses gene expression by delivering mRNA cargo to processing bodies (P-bodies) where the mRNA is degraded. TTP functions as a tumor suppressor in a mouse model of B cell lymphoma, and in some human malignancies low *TTP* expression correlates with reduced survival. Here we report important prognostic and functional roles for TTP in human prostate cancer. First, gene expression analysis of prostate tumors revealed low *TTP* expression correlates with patients having high-risk Gleason scores and increased biochemical recurrence. Second, in prostate cancer cells with low levels of endogenous TTP, inducible TTP expression inhibits their growth and proliferation, as well as their clonogenic growth. Third, TTP functions as a tumor suppressor in prostate cancer, as forced TTP expression markedly impairs the tumorigenic potential of prostate cancer cells in a mouse xenograft model. Finally, pathway analysis of gene expression data suggested metabolism is altered by TTP expression in prostate tumor cells, and metabolic analyses revealed that such processes are impaired by TTP, including mitochondrial respiration. Collectively, these findings suggest that TTP is an important prognostic indicator for prostate cancer, and augmenting TTP function would effectively disable the metabolism and proliferation of aggressive prostate tumors.

## INTRODUCTION

Tristetraprolin (TTP, ZFP36) is an RNA-binding protein that contains tandem CCCH zinc finger domains that bind to adenosine-uridine (AU)-rich elements located in the 3′ untranslated region (3′UTR) of many short-lived mRNAs [1]. AU-rich elements are found in numerous genes involved in tumorigenesis, including those encoding proteins that control proliferation, metabolism, immune responses, angiogenesis, and metastasis [2]. Once TTP binds to these labile mRNAs, they relocate to processing bodies (P-bodies) where the mRNA is degraded by a series of RNA decay enzymes [3]. Alterations in TTP levels can have serious biological consequences. For example, the absence of TTP in knockout mice leads to marked increases in *tumor necrosis factor-α* (*TNFα*) mRNA, which provokes a severe autoimmune disease marked by runting, cachexia, arthritis, and dermatitis [4].

Notably, several lines of evidence indicate that TTP can have profound effects on tumor growth and development. First, in the Eμ-*Myc* transgenic mouse model of B cell lymphoma, TTP functions as a tumor suppressor that impairs the development of lymphoma, and disables maintenance of the malignant state [5]. Second, in colon cancer silencing TTP provokes increases in the expression of *COX-2* and *VEGF*, which enhance colon tumorigenesis [6, 7]. Third, *TTP* is commonly lost in cervical cancer and this is linked to the destruction of the p53 tumor suppressor, as TTP binds to and destabilizes the mRNA encoding the ubiquitin ligase E6-AP [8]. Fourth, in breast cancer depletion of *TTP* augments levels of IL-16, which promotes monocyte and macrophage tumor infiltrates and tumor progression [9]. Fifth, low TTP levels in mutant *BRAF^V600E^*-driven melanoma triggers increased levels of IL-8 (CXCL8), tumor growth, and angiogenesis [10]. Finally, low TTP levels in human pancreatic cancer, breast cancer, and lung adenocarcinoma correlate with advanced tumor stage and reduced survival rates, suggesting that low TTP levels are a poor prognostic indicator for cancer patients [11, 12].

In the United States prostate cancer is the most common non-skin malignancy and the second leading cause of cancer deaths in men [13]. However, most men diagnosed with prostate cancer will not die from this disease. Thus, there is a critical need to define the molecular differences between indolent and aggressive prostate tumors. Previous analyses of a human prostate cancer dataset have suggested that metastatic tumors express lower levels of *TTP* than primary tumors [14]. Further, a gene signature generated using The Cancer Genome Atlas (TCGA) for tumors with low *TTP* expression shares significant similarities to the genes that are differential expressed between prostate cancer patients with higher-risk Gleason score 8-10 tumors versus those with lower-risk Gleason score 5-6 tumors [11].

Given these findings we hypothesized that low levels of TTP expression are required for aggressive prostate cancer growth and proliferation. Indeed, here we report that low *TTP* mRNA levels are a poor prognostic indicator for primary prostate cancer patients. Further, TTP functions as a tumor suppressor that impairs prostate cancer cell growth *ex vivo* and tumorigenicity *in vivo*. Finally, the tumor suppressor functions of TTP are linked to marked alterations in prostate cancer cell metabolism. Thus, TTP is a potential biomarker and critical regulator of prostate cancer.

## RESULTS

### Low *TTP* expression is a poor prognostic indicator for human prostate cancer patients

**Figure 1:**
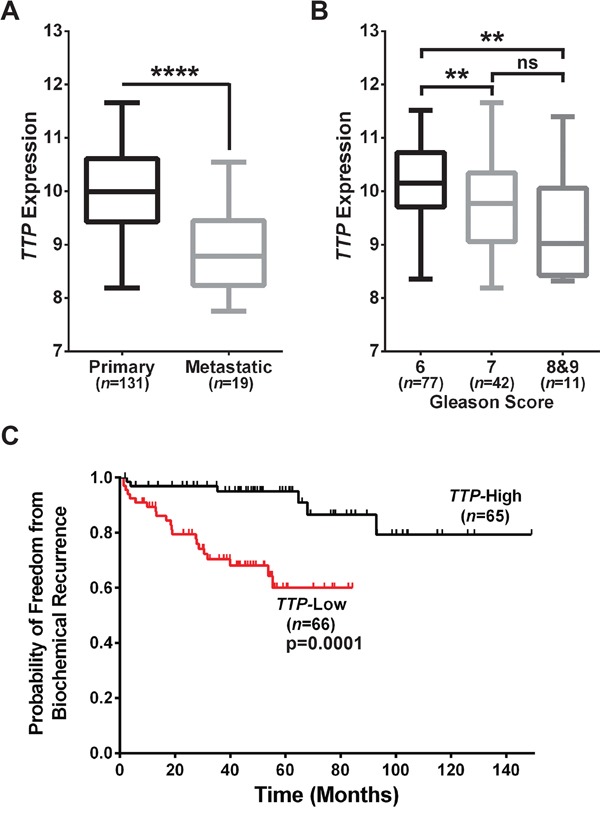
Low *TTP* expression connotes poor prognosis for human prostate cancer patients **A.** Comparison of *TTP* expression in primary versus metastatic prostate tumors (****p<0.0001, Mann-Whitney test). **B.** *TTP* expression in tumors from Gleason Score 6, Gleason Score 7, and Gleason Scores 8&9 primary prostate cancer patients (**p<0.01, Mann-Whitney test; ns, not significant). **C.** Analysis of biochemical recurrence in primary prostate cancer patients separated into *TTP*-High and *TTP-*Low cohorts based on median *TTP* expression levels (p=0.0001, Mantel-Cox log-rank test). Data provided in GEO Dataset GSE21034 [15] was used for all of the analyses in this figure. Error bars in (A) and (B) indicate maximum to minimum values for all samples in each group.

To determine if reduced levels of *TTP* mRNA might be a hallmark of aggressive prostate cancers we initially analyzed a gene expression dataset of 131 primary and 19 metastatic human prostate cancer samples (GSE21034) [15]. Indeed, these analyses revealed that metastatic prostate tumors have lower *TTP* levels than primary tumors (Figure 1A), in accord with findings of others using a different prostate cancer dataset [14]. As part of standard prostate cancer prognosis, patients undergo a tumor biopsy that is examined and graded by a pathologist to determine the tumor stage and Gleason score, which provides a general indication of the risk that the tumor poses to the overall health of the patient. Analysis of *TTP* expression in the primary tumors in this dataset revealed that high-risk Gleason score 8 and 9 prostate tumors, as well as intermediate-risk Gleason score 7 tumors, have lower levels of *TTP* than low-risk Gleason score 6 prostate tumors (Figure 1B). Finally, the patients with primary prostate cancer were separated into two groups, *TTP-*High and *TTP*-Low, based on the median *TTP* expression level in this dataset. These two cohorts were then analyzed for their rate of biochemical recurrence (BCR), an early indication that prostate cancer might relapse, which is defined in the GSE21034 dataset as PSA ≥ 0.2 ng/ml on two occasions. Patients in the *TTP-*Low cohort had a substantially increased rate of BCR compared to *TTP*-High patients (Figure 1C), suggesting that *TTP*-Low patients might have an increased risk of tumor relapse. Collectively, these studies show that low *TTP* expression in prostate cancer is associated with more aggressive tumors, and they suggest that *TTP* is potentially an important diagnostic biomarker for prostate cancer.

### TTP impairs the growth and proliferation of prostate cancer cells

**Figure 2:**
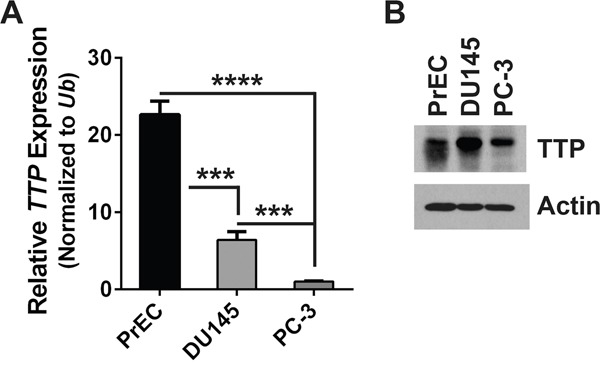
PC-3 cells are a model of human prostate tumors that express low levels of TTP **A.** qRT-PCR analysis of *TTP* expression in primary prostate epithelial cells (PrEC) and prostate cancer cell lines DU145 and PC-3. Results were normalized to levels of *Ubiquitin* (*Ub*) mRNA. Standard error bars are provided (*n*=3; ***p<0.001, ****p<0.0001, Student's t-test). **B.** Immunoblot blot analyses of TTP and Actin protein levels in PrEC, DU145, and PC-3 cells.

Primary prostate epithelial cells (PrEC) and two human prostate cancer cell lines, DU145 and PC-3, were compared for levels of TTP mRNA and protein. Quantitative real-time PCR (qRT-PCR) analysis found that PrEC express significantly higher levels of TTP than the two prostate cancer cell lines (Figure 2A). In addition, PC-3 cells express lower levels of TTP mRNA and protein than PrEC and DU145 cells (Figure 2A and 2B). Thus, PC-3 cells provide an *in vitro* model for prostate cancer with low *TTP* expression.

**Figure 3:**
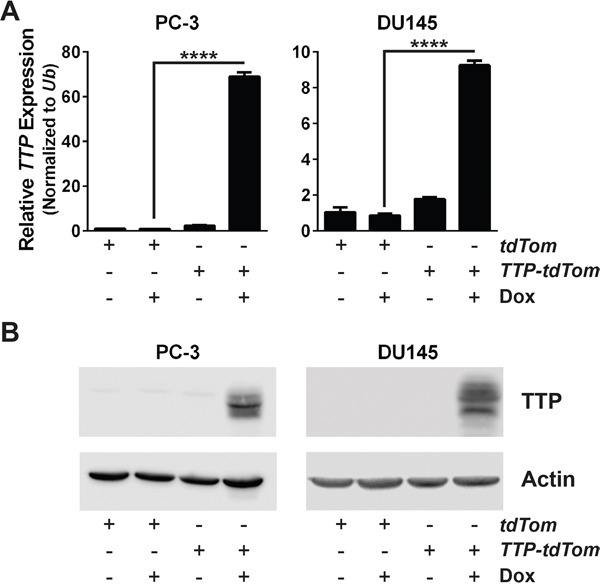
An inducible expression system increases TTP RNA and protein levels in prostate cancer cells **A.** qRT-PCR analysis of *TTP* mRNA levels in PC-3 or DU145 cells expressing *rtTA^2^* + *tdTom* or *rtTA^2^* + *TTP* + *tdTom* either untreated or Dox-treated for 48 hr. Results were normalized to levels of *Ub* (*n*=3; ****p<0.0001, Student's t-test). **B.** Immunoblot analyses of TTP and Actin levels in PC-3 or DU145 cells expressing *rtTA^2^* + *tdTom* or *rtTA^2^* + *TTP* + *tdTom* either untreated or Dox-treated for 48 hr.

In lymphomas arising in the Eμ-*Myc* transgenic mouse, a model of human B cell lymphomas with *MYC* involvement, expression of TTP provokes growth arrest [5]. To test the biological effects of TTP in prostate cancer, PC-3 cells were transduced with a reverse tetracycline-inducible (Tet-On) expression system, to allow for inducible TTP expression following the addition of doxycycline (Dox) to the cell culture media. First, PC-3 cells were infected with a pRetroX-Tet-On-Advanced retrovirus that contains the gene for the advanced reverse tetracycline transactivator (*rtTA^2^*), and infected cells were selected for by culturing in G418 containing medium. Then, PC-3-expressing rtTA^2^ cells were infected with either a pRetroX-Tight-pPGK-*tdTomato* (*tdTom*) or a pRetroX-Tight-*TTP*-pPGK-*tdTomato* (*TTP-tdTom*) retrovirus, and transduced cells were sorted by flow cytometry for tdTom expression. To confirm that differences found using this system were generalizable effects of TTP, DU145 cells were also transduced to have Dox-inducible expression of TTP using the same rtTA^2^ expression system described above for PC-3 cells. Following expansion in culture, both of these prostate cancer cell lines were tested for their ability to induce TTP expression following Dox treatment. Notably, Dox treatment of PC-3 and DU145 cells engineered to express rtTA^2^ + TTP both displayed a robust induction of both TTP mRNA and protein (Figure 3A and 3B).

**Figure 4:**
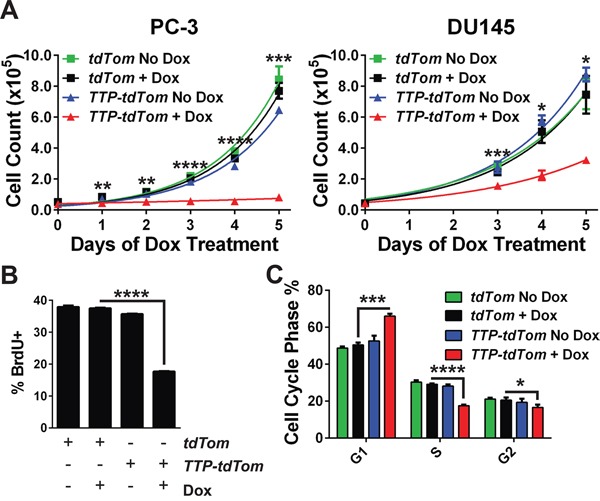
TTP impairs prostate cancer cell growth **A.** Growth of PC-3 or DU145 cells expressing *rtTA^2^* + *tdTom* or *rtTA^2^* + *TTP* + *tdTom* after seeding in medium containing Dox. The average number of cells for each cell line was determined daily (*n*=3; **p<0.01, ***p<0.001, ****p<0.0001, Student's t-test). **B.** Proliferation of PC-3 cells expressing *rtTA^2^* + *tdTom* or *rtTA^2^* + *TTP* + *tdTom* that were untreated or Dox-treated for 48 hr, was determined by pulse-labeling with BrdU and flow cytometry analyses (*n*=3; ****p<0.0001, Student's t-test). **C.** Cell cycle distribution of PC-3 cells expressing *rtTA^2^* + *tdTom* versus *rtTA^2^* + *TTP* + *tdTom* was determined 48 hours after the addition of Dox. Cells were stained with DAPI and analyzed by flow cytometry (*n*=3; *p<0.05, ***p<0.001, ****p<0.0001, Student's t-test). Error bars provided indicate standard error.

To assess potential effects of TTP on the growth of prostate cancer cells, growth curve assays were performed in triplicate for both cell lines. These analyses showed that the induction of TTP expression led to an immediate cessation of PC-3 cell growth and a significant impairment of DU145 cell growth (Figure 4A). The decrease in PC-3 cell growth was associated with decreased rates of DNA synthesis as shown by the reduction of BrdU positive cells following TTP induction (Figure 4B), and cell cycle analyses indicated that growth arrest was associated with an increased percentage of cells in G_1_ phase and a corresponding decrease of cells in the S and G_2_ phases (Figure 4C). However, the apoptotic index was not augmented by the induction of TTP in either prostate cancer cell line (Supplementary Figure S1). Further, TTP-induced growth arrest was not due to the induction of senescence as cells removed from Dox treatment resumed their normal rates of growth, and there were no increases in β-galactosidase activity in PC-3 cells following TTP induction (Supplementary Figure S2A and S2B). Thus, as observed in mouse B cell lymphomas [5], TTP disables maintenance of the malignant state of prostate cancer cells by provoking a growth arrest response.

### TTP impairs the tumorigenicity of prostate cancer cells

**Figure 5:**
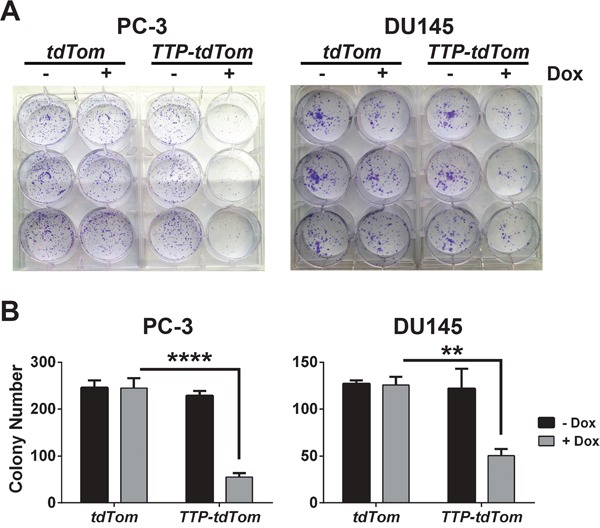
TTP impairs the clonogenic growth of prostate cancer cells **A.** Clonogenic growth assay of PC-3 or DU145 cells expressing *rtTA^2^* + *tdTom* or *rtTA^2^* + *TTP* + *tdTom* cells +/− Dox. **B.** Average number of colonies per well for each condition (*n*=3) of the clonogenic growth assay in (A) is shown (*n*=3; **p<0.01, ****p<0.0001, Student's t-test). Error bars indicate standard error.

**Figure 6:**
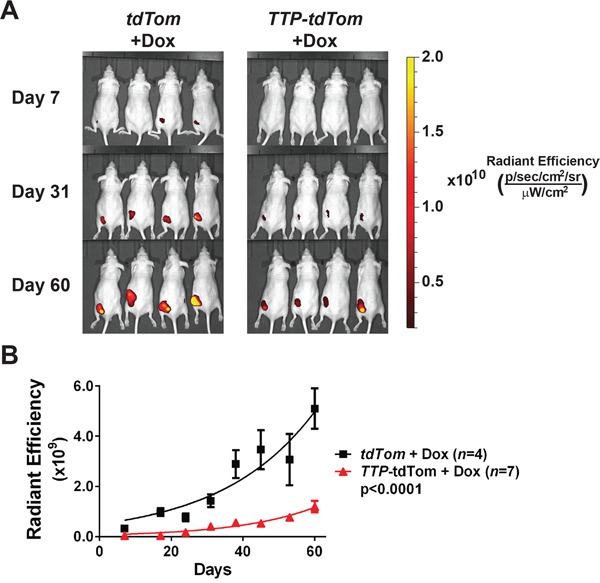
TTP impairs the tumorigenicity of PC-3 cells **A.** Fluorescence images of tumor xenografts in *Nude* mice injected subcutaneously with PC-3 cells expressing *rtTA^2^* + *tdTom* or *rtTA^2^* + *TTP* + *tdTom*. All mice were switched to diet containing Dox three days after transplant. **B.** Average tumor size (radiant efficiency [(p/sec/cm^2^/sr)/(μW/cm^2^)]) was measured weekly for tumor xenografts shown in (A) (p<0.0001, two-way ANOVA). Error bars indicate standard error.

To test if TTP would impair the tumorigenic potential of prostate tumor cells to grow as clonogenic colonies *ex vivo*, PC-3 and DU145 cells expressing rtTA^2^ + tdTom or rtTA^2^ + TTP + tdTom were each plated at low density and observed for colony formation. In both PC-3 and DU145 cells expressing rtTA^2^ the ability to form clonogenic colonies was unaffected by the addition of Dox. In contrast, TTP induction markedly hindered the clonogenic potential of PC-3- and DU145-expressing rtTA^2^ + TTP + tdTom cells (Figure 5A and 5B). Furthermore, to test if TTP expression would impair tumorigenicity *in vivo*, PC-3 cells expressing rtTA^2^ + tdTom or those expressing rtTA^2^ + TTP + tdTom were subcutaneously injected into *Nude* mice. After 3 days, the mice were switched to a diet containing Dox and tumor progression was monitored by IVIS imaging for tdTom. PC-3 tumors expressing rtTA^2^ + tdTom grew rapidly. In contrast, PC-3 tumors expressing rtTA^2^ + TTP + tdTom had significantly reduced rates of tumor growth (Figure 6A and 6B). Thus, TTP is a tumor suppressor of prostate cancer.

### TTP impairs the metabolic functions of prostate cancer cells

**Figure 7:**
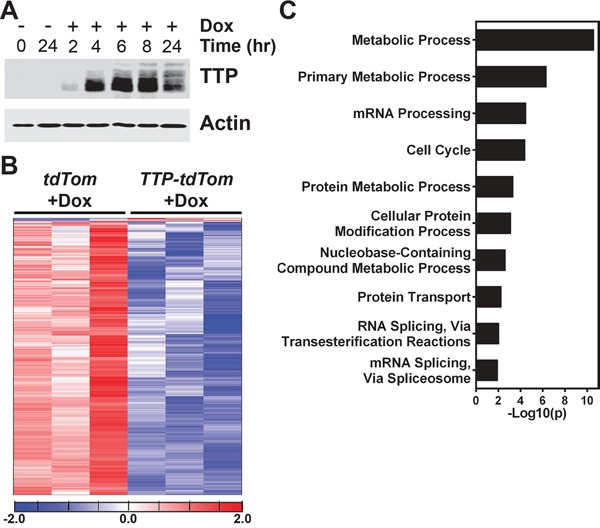
TTP alters metabolic processes in PC-3 cells **A.** Immunoblot analyses of TTP and Actin levels in PC-3 cells expressing *rtTA^2^* + *TTP* + *tdTom* either untreated or Dox-treated for the times indicated. **B.** Gene expression profiling of RNA-Seq data revealed that 1755 genes were differentially expressed between PC-3 cells expressing *rtTA^2^* + *tdTom* or *rtTA^2^* + *TTP* + *tdTom* after 4 hours of Dox treatment. Differentially expressed genes are provided in Supplementary Table S1. **C.** PANTHER analysis results showing the ten gene ontologies with the highest gene enrichment values for the genes differentially expressed between Dox-treated (4 hr) PC-3 cells expressing *rtTA^2^* + *tdTom* or *rtTA^2^* + *TTP* + *tdTom*.

To gain insights into the mechanism by which TTP blocks prostate cancer cell growth we performed RNA sequencing (RNA-Seq) analyses. To determine the earliest time that TTP protein could be detected in these cells, PC-3 cells expressing rtTA^2^ + TTP + tdTom cells were treated with Dox across a time course. Immunoblotting determined that TTP protein was readily detected by four hours post-Dox treatment (Figure 7A). RNA was then collected from rtTA^2^ + tdTom- or rtTA^2^ + TTP + tdTom-expressing PC-3 cells treated with Dox for four hours for RNA-Seq analysis. The RNA-Seq gene expression data from these two cell lines were compared, and 1755 unique gene transcripts were identified as being altered by TTP induction in PC-3 cells (Figure 7B and Supplementary Table S1). Almost all (~99%) of these genes had reduced expression levels in the PC-3 cells expressing TTP, as expected since TTP destabilizes mRNA expression [3]. Furthermore, this list of mRNAs is enriched for those previously identified as being able to be bound by TTP [16] (640 genes; p<1e^−5^, Fisher's exact test) and to have AU-rich elements in their 3′UTR [2] (597 genes; p<1e^−5^, Fisher's exact test). In total, 984 individual mRNAs altered by TTP expression in PC-3 cells have either previously been shown to be bound by TTP or have AU-rich elements, and 253 of these mRNAs have been shown to both be bound by TTP and have AU-rich elements. This suggests that many of these transcripts may be post-transcriptionally regulated by TTP.

**Figure 8:**
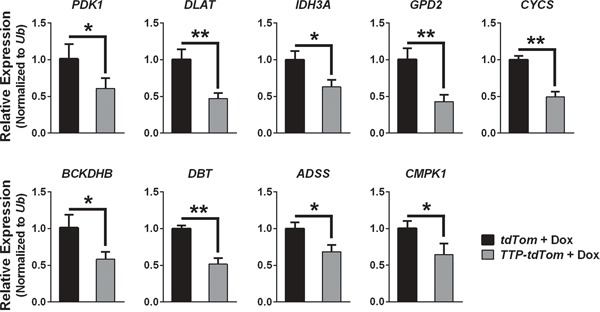
TTP alters the expression of genes involved in metabolic pathways in PC-3 prostate cancer cells qRT-PCR analyses showing the RNA expression levels of the indicated metabolic genes in Dox-treated (4 hr) PC-3 cells expressing *rtTA^2^* + *tdTom* or *rtTA^2^* + *TTP* + *tdTom*. qRT-PCR results were normalized to levels of *Ub*. Error bars indicate standard error (*n*=3; *q<0.05, **q<0.01, Student's t-test corrected for multiple testing).

The genes differentially expressed by TTP induction in the RNA-Seq analysis were analyzed using the PANTHER GO-Slim Biological Process Classification System [17] to identify which biological pathways might be affected by TTP induction in prostate cancer cells. Unexpectedly, PANTHER analysis found that the metabolic process and primary metabolic process gene ontologies were the most significantly enriched by TTP expression suggesting that metabolic pathways are regulated by TTP (Figure 7C). A subset of nine genes found to be differential expressed in the RNA-Seq data from the primary metabolic process gene ontology were selected for further analysis by qRT-PCR to confirm that their expression is altered by the induction of TTP in PC-3 cells. These genes were selected to represent multiple metabolic pathways, including the pyruvate dehydrogenase complex (*PDK1*, *DLAT*), the citric acid cycle (*IDH3A*), the electron transport chain (*GPD2*, *CYCS*), branched-chain amino acid metabolism (*BCKDHB*, *DBT*), purine biosynthesis (*ADSS*), and the pyrimidine salvage pathway (*CMPK1*). Furthermore, with the exception of *PDK1*, these genes have been previously shown to either be bound by TTP [16] and/or to have AU-rich elements in their 3′UTRs [2] (Supplementary Table S1). These analyses confirmed that the expression of these metabolic genes was indeed reduced in Dox-treated TTP + tdTom-expressing PC-3 cells compared to Dox-treated tdTom-expressing PC-3 cells (Figure 8); however, RNA immunoprecipitation studies must be performed to prove that TTP directly binds to and regulates these mRNAs in prostate cancer cells.

RNA-Seq analysis also revealed that TTP affects genes involved in other mechanisms important for prostate cancer growth and progression, and qRT-PCR confirmed these genes were down-regulated by TTP. This cast of targets includes oncogenes (*KRAS*, *ETS2*), cell cycle genes (*CCNC*, *CDK1*), metastasis genes (*ADAM9*), inflammatory chemokines (*CXCL1*, *IL8*), and subunits of PKC (*PRKAR1A*, *PRKACB*) (Supplementary Figure S3). Altogether, these studies suggest that TTP is a critical regulator of genes involved in prostate cancer development, progression, and metastasis.

**Figure 9:**
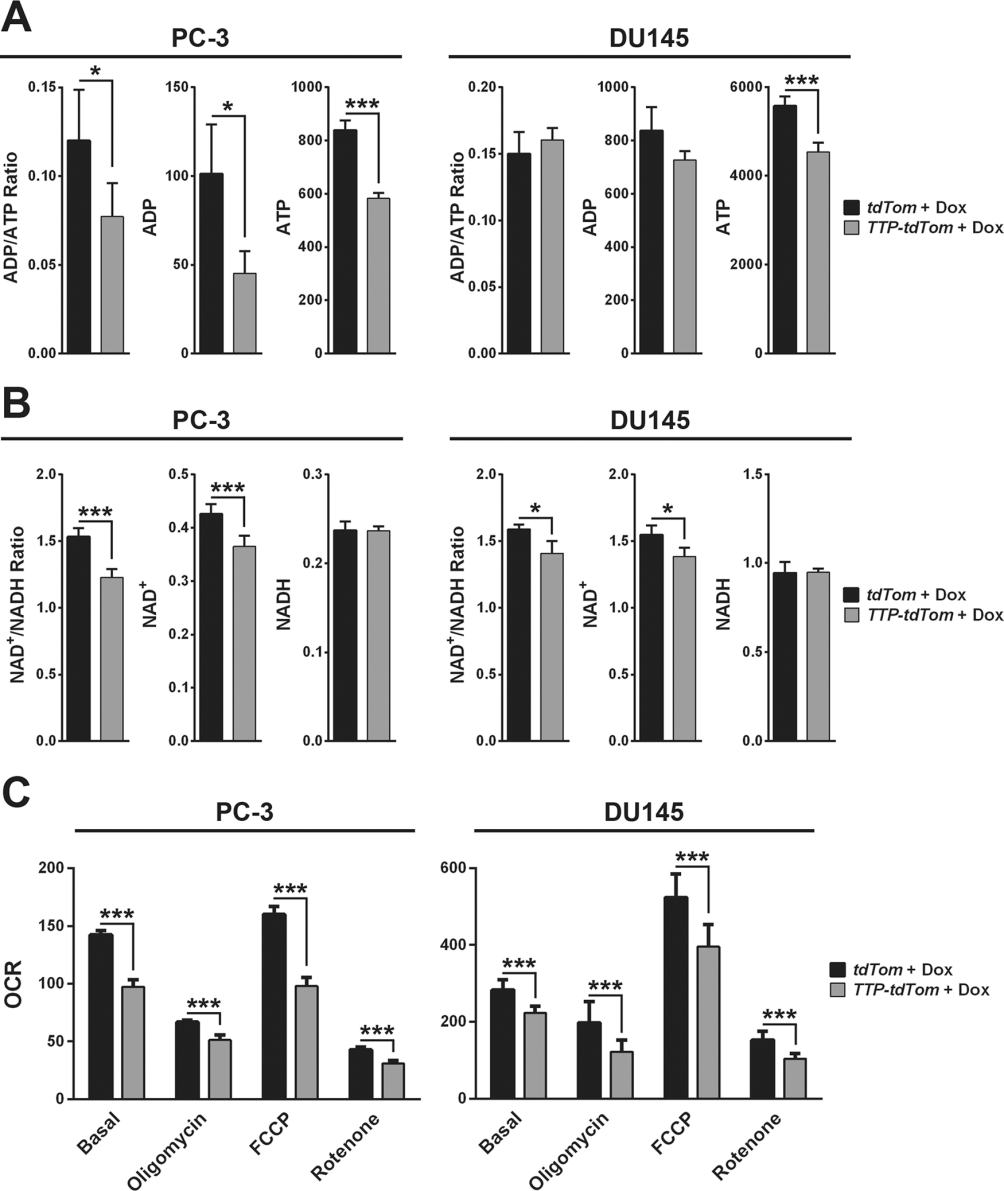
TTP impairs the metabolic functions of prostate cancer cells **A.** Bioluminescent assay of ADP and ATP levels (relative light units/μg protein) in Dox-treated (4 hr) PC-3 or DU145 cells expressing *rtTA^2^* + *tdTom* or *rtTA^2^* + *TTP* + *tdTom* (*n*=3; *p<0.05, ***p<0.001, Student's t-test). **B.** Colorimetric assay of NAD+ and NADH levels (pmol/μg protein) in Dox-treated (4 hr) PC-3 or DU145 cells expressing *rtTA^2^* + *tdTom* or *rtTA^2^* + *TTP* + *tdTom* (*n*=3; *p<0.05, ***p<0.001, Student's t-test). **C.** Seahorse analysis of OCR (pmol/minute) in Dox-treated (4 hr) PC-3 or DU145 cells expressing *rtTA^2^* + *tdTom* or *rtTA^2^* + *TTP* + *tdTom*. Basal rates, and rates following the addition of oligomycin, FCCP, or rotenone, are shown (*n*=6; ***p<0.001, Student's t-test). Error bars provided indicate standard error.

To confirm that down-regulation of metabolic genes by TTP has functional consequences, the levels of select metabolites were measured in PC-3 or DU145 cells expressing rtTA^2^ + tdTom versus those expressing rtTA^2^ + TTP + tdTom following four hours of Dox treatment. First, the levels of ATP were decreased following TTP induction in both PC-3 and DU145 cells, and ADP levels were also lowered by TTP in PC-3 cells (Figure 9A). Reductions in ATP levels indicate that TTP compromises overall energy production in prostate cancer cells, and marked reductions in ADP levels show that the substrate required to make more ATP is also altered. Second, the levels of NAD+, a required cofactor for many redox reactions in metabolism, were also reduced in both cell lines, suggesting that metabolism is immediately dampened following TTP induction (Figure 7B). In contrast, the levels of NADP^+^ and NADPH forms were not altered by TTP in prostate cancer cells (Supplementary Figure S4). Finally, the oxygen consumption rate (OCR) and extracellular acidification rate (ECAR) were measured in both of these cell lines after the addition of Dox. The basal level of OCR, the level of OCR following treatment of cells with oligomycin A, which blocks ATP synthase, the maximal level of OCR, measured following treatment of cells with the mitochondrial uncoupling agent FCCP, and level of OCR following treatment with rotenone, which blocks electron transport, were all markedly reduced following acute TTP induction (four hours post-Dox treatment) in both PC-3 and DU145 cells (Figure 9C and Supplementary Figure S5A and S5B). In addition, OCR levels in DU145 cells were further decreased in all treatment conditions when the cells were treated with Dox for 20 hours, suggesting that prolonged TTP induction enhances the impairment of mitochondrial respiration (Supplementary Figure S5C). In contrast, ECAR is unchanged by TTP in all of the conditions measured suggesting that TTP does not affect glycolysis in prostate tumor cells (Supplementary Figure S6). Thus, the tumor suppressor functions of TTP in prostate cancer include impairing oxidative phosphorylation, which is necessary for prostate tumor growth.

## DISCUSSION

The data presented herein establish that the mRNA-binding and destabilizing protein TTP plays important roles in harnessing prostate cancer growth and tumorigenicity, and they suggest that reactivating TTP expression in aggressive prostate tumors, which express low levels of this protein, would be an effective means for disabling tumor metabolism. Specifically, using a tetracycline-inducible system to activate TTP expression in metastatic PC-3 prostate cancer cells that express low levels of endogenous TTP, we found that TTP blocks proliferation and provokes cell cycle arrest *ex vivo* and impairs tumorigenicity *in vivo*. Furthermore, using the same system to induce TTP in another metastatic prostate cancer cell line, DU145, confirmed many of these findings. In addition, these results are similar to those that established TTP as a tumor suppressor in Myc-driven mouse B cell lymphoma, where this protein also provokes cell cycle arrest *ex vivo* [5]. They are also generally in accord with the *in vitro* finding of others showing that prostate cancer cell proliferation can be altered by manipulating TTP levels [18], though our findings in PC-3 cells show that this does not occur through TTP targeting E2F1 expression (Supplementary Figure S7).

Importantly, the data presented here establish that TTP functions as a tumor suppressor of prostate cancer. First, induction of TTP expression in PC-3 and DU145 prostate cancer cells impairs their ability to form colonies *ex vivo*. Further, activation of TTP in PC-3 cells blocks their ability to grow *in vivo* as subcutaneous xenografts. These results are in accord with those performed in the Myc-driven B cell lymphoma mouse model where enforced TTP expression in B cells disables the maintenance of lymphomas in syngeneic transplant model [5]. Interestingly, a recent study reported links of histone deacetylase inhibitor (HDACi) activity to TTP, where treatment of colorectal cancer cells with HDACi activates the EGR1 transcription factor to induce TTP and provoke growth arrest [19]. Collectively, these findings suggest that developing new therapeutics that specifically stimulate TTP expression may be an avenue for treating patients with low-TTP expressing tumors.

Further, RNA-Seq analyses were performed to determine which biological pathways are directly affected by TTP expression in prostate cancer. To minimize secondary effects of TTP induction, these analyses were from cells treated with Dox for only four hours, which was the earliest time post-Dox treatment that TTP protein was present. Notably, over 1700 genes were still identified as having their expression altered in response to TTP. These results are similar to studies evaluating TTP's role in Myc-driven B cell lymphoma, where the expression of ~1100 genes was altered following acute TTP expression in *ex vivo* lymphoma cells [5]. Surprisingly, pathway analysis of genes differentially expressed by TTP induction in prostate cancer cells identified those involved in metabolic processes, in particular mitochondrial respiration and ATP homeostasis, as being the most commonly altered by TTP. Included were genes that express enzymes involved in the pyruvate dehydrogenase complex (*PDK1, DLAT*), the citric acid cycle (*IDH3A*), the electron transport chain (*GPD2*, *CYCS*), branched-chain amino acid catabolism (*BCKDHB*, *DBT*), purine biosynthesis (*ADSS*), and pyrimidine salvage (*CMPK1*). Most importantly, functional studies confirmed that the induction of TTP rapidly reduces both mitochondrial respiration and energy stores in prostate cancer cells. To our knowledge this is the first report of TTP regulating cancer metabolism. Notably, re-evaluation of expression profiling data following acute TTP expression in Myc-driven B cell lymphoma [5] found that mRNAs encoding the metabolic enzymes *Dlat*, *Idh3a*, *Gpd2*, and *Cycs* are also rapidly down-regulated targets of TTP in this context (data not shown). Thus, TTP impairs the expression of genes involved in mitochondrial respiration in both prostate cancer and lymphoma, and this is a key biological pathway altered by TTP in cancer. Further studies will be performed in both tumor models to understand how TTP affects cancer metabolism.

Finally, a major clinical challenge for prostate cancer treatment is the need to reduce overtreatment of this disease, where it has been estimated that in the United States two out of three low-risk prostate cancer patients receive unwarranted therapy [20]. This unnecessary treatment and its debilitating side effects, including incontinence, erectile and bowel dysfunction, severe pain, hot flashes, and muscle weakness, negatively impact the quality of life for tens of thousands of men annually. One way to address this challenge is to identify new biomarkers that distinguish aggressive from indolent prostate cancer, which would allow physicians to determine appropriate treatment plans that improve the quality of life for prostate cancer patients. Excitingly, the studies presented here reveal that *TTP* mRNA is an exceptionally promising biomarker candidate for prostate cancer risk assessment. First, there are marked reductions of *TTP* mRNA levels in metastatic prostate cancer compared to primary tumors. Further, it was found that men with low *TTP*-expressing primary prostate cancer have significantly increased chances of biochemical recurrence, an early precursor for tumor recurrence. These data strongly support the notion that TTP is also a promising prognostic biomarker that distinguishes aggressive versus indolent prostate cancer, which warrants future investigation.

## MATERIALS AND METHODS

### Cell culture

Human primary prostate epithelial cells (PrEC) (Lonza, CC-2255, Basel, Switzerland) were grown in the Prostate Epithelial Growth Media BulletKit (Lonza, CC-3166) according to the manufacturer's protocol. Human prostate cancer cell lines DU145 (ATCC, HTB-81, Manassas, VA, USA) and PC-3 (ATCC, CRL-1435) were maintained according to the manufacturer's protocol.

The Retro-X Tet-On Advanced Inducible Expression System (Clontech, 632104, Mountain View, CA, USA) was used to transduce PC-3 and DU145 cells with a reverse tetracycline transactivator (*rtTA^2^*). Briefly, 293T cells were CaPO_4_ transfected (Promega, E1200, Madison, WI, USA) with pRetroX-Tet-On-Advanced (*rtTA^2^*), pRetroX-Tight-pPGK-*tdTomato* (*tdTom*), or pRetroX-Tight-*TTP*-pPGK-*tdTomato* (*TTP-tdTom*) plus pMD1-old-*gag pol* and pCMV-VSV-G to produce retroviral supernatants. After filtration, retrovirus with polybrene was added to PC-3 or DU145 cells. Following infection with *rtTA^2^* retrovirus, cells were selected with G418 treatment. PC-3 + *rtTA^2^* and DU-145 + *rtTA^2^* cells were tested using the pRetroX-Tight-Pur-*Luc* plasmid in a Dox gradient, and maximal luciferase induction was found to occur with 300 ng/ml Dox treatment, which was used for all *in vitro* experiments presented. Then cells were infected with a second retrovirus containing either pRetroX-Tight-pPGK-*tdTomato* (*tdTom*), or pRetroX-Tight-*TTP*-pPGK-*tdTomato* (*TTP-tdTom*). Following infection with *tdTom* or *TTP-tdTom* cells were sorted by flow cytometry.

For growth curve analysis, 4x10^4^ cells were seeded overnight before Dox treatment. Three wells of each condition were individually collected at selected intervals and viable cells were counted. Media and Dox were replaced 72 hr after initial Dox treatment for growth curves longer than three days.

For apoptosis analysis, 2,500 cells were seeded in a 96 well plate overnight before Dox treatment. After the addition of Dox, three wells of each condition (No Dox and Dox-treated) were grown for 48 hours. Then using the Caspase-Glo 3/7 Assay (Promega, G8090, Madison, WI, USA) the luminescence produced by active Caspase 3 and Caspase 7 in each well was measured on a BioTek Cytation 3 imaging microplate reader (BioTek, Winooski, VT, USA). For clonogenic growth assays, 800 cells were seeded 48 hr before Dox treatment and assay was performed as previously described [21].

### RNA preparation and analyses

RNA was prepared, cDNA was synthesized, and quantitative real-time PCR (qRT-PCR) was performed as previously described [5]. Data analyses used the ΔΔCt method, where levels of *ubiquitin* (*Ub*) mRNA served as the internal control. For experiments where the expression of multiple genes was tested, we generated q-values using a correction for multiple testing as described by Benjamini and Hochberg [22]. Oligonucleotides are provided in Supplementary Table S2.

### Western blot analyses

Protein from cells was prepared, separated on SDS-PAGE gels, and transferred to membranes as previously described [5]. Membranes were blotted for antibodies specific for TTP (from Dr. Perry Blackshear, National Institute of Environmental Health Sciences, Research Triangle Park, NC, USA), E2F1 (BD Transduction Labs, E511120, San Diego, CA, USA) and actin (Sigma, AC-15, St. Louis, MO, USA).

### Gene expression profiling analysis

Normalized gene expression data was obtained from GSE21034 [15] and converted to log2 expression. Statistical analysis of microarray gene expression was performed using a two-tailed Mann-Whitney test for group comparisons, and statistical analysis of biochemical recurrence data was performed using a log-rank test, both using GraphPad Prism 6.0d (GraphPad Software Inc., La Jolla, CA, USA).

RNA was prepared from Dox-treated cells (4 hr) to generate RNA Sequencing (RNA-Seq) libraries. RNA-Seq library preparation was performed using the NuGen Ovation Encore Complete kit. Briefly, 100 ng of RNA was used to generate cDNA and a strand-specific library following the manufacturer's protocol (NuGEN Technologies, Inc., San Carlos, CA, USA). Quality control steps including analysis on the BioAnalyzer RNA chip and qRT-PCR for library quantification were performed. The libraries were then sequenced on the Illumina NextSeq 500 sequencer (Illumina, San Diego, CA, USA) with a 75-base paired-end run in order to generate 40-50 million read pairs per sample. Sequence reads were aligned to the human genome (Hs37D5) in a splice-aware fashion using Tophat2 software [23]. Aligned reads were then condensed into transcripts to calculate differential expression at both the gene and transcript levels with Cufflinks software [24]. Cufflinks FPKM values of differential expressed genes were used to generate heatmaps using MATLAB R2014b software (The MathWorks Inc.). The RNA-Seq data for these samples are available from the Gene Expression Omnibus (GEO) under Accession GSE87532. Gene over-representation analysis was performed using the PANTHER GO-Slim Biological Process annotation dataset in the PANTHER Classifications (Version 10.0) [17] and a Bonferroni corrected p-value.

### Flow cytometry analysis

Dox-treated cells (48 hr) were analyzed using the FITC BrdU Flow Kit (BD Biosciences, 559619, San Diego CA, USA) as previously described [5].

For cell cycle analysis, Dox-treated cells (48 hr) were harvested and fixed overnight in 70% ethanol at 4°C. Fixed cells were then washed twice in PBS and stained with 1.25 μg/ml DAPI (Sigma, D9542). Cells were sorted using a FACSCanto II flow cytometer and the data was analyzed using a ModFit*LT* manual deconvolution model (Verity Software House, Topsham, ME, USA).

For senescence analysis, Dox-treated cells (48 hr) were pre-treated with 50 μM chloroquine for 1 hr to increase the internal pH of lysosomes, and were then stained with 33 μM of 5-dodecanoylaminofluorescein di-β-D-galactopyranoside (C_12_FDG), a fluorogenic substrate for β-galactosidase activity [25]. Cells were sorted using an LSR II flow cytometer (BD Biosciences) and senescence analysis was performed using Flowjo software (Flowjo LLC, Ashland, OR, USA). For all flow cytometry analyses, aggregates were gated out using forward and side scatter pulse width gating, and the data were analyzed using singlets.

### Xenograft assay

PC-3 cells expressing *rtTA^2^* + *tdTom* or *rtTA^2^* + *TTP* + *tdTom* were resuspended in PBS, and mixed 1:1 with matrigel (BD Biosciences, 356234) at 1x10^7^ cells/ml. 1x10^6^ cells (100 μl) were injected subcutaneously into the left rear flank of 8 week old male *Nude* recipients (The Jackson Laboratory, 002019, Bar Harbor, ME, USA). Three days after transplant the mice were changed to a diet containing Dox (1g/kg) (Bio-Serv, S3949, Flemington, NJ, USA)). Mice were imaged using an IVIS 200 system (PerkinElmer, Waltham, MA, USA) to measure the tumors. Radiant efficiency [(photons/sec/cm^2^/sr)/(μW/cm^2^)] for each tumor was calculated by Living Image 3.1 software (PerkinElmer) and averaged for each PC3 xenograft subtype. All animal studies were approved by the University of South Florida IACUC.

### Metabolic assays

Cells were seeded overnight and Dox treated for either 4 hr or 20 hr as indicated in the text. ADP/ATP, NADP^+^/NADPH, NAD^+^/NADH ratios were measured using detection kits (Sigma-Aldrich, MAK135, MAK038, and MAK037, respectively). Oxygen consumption rate (OCR) and extracellular acidification rate (ECAR) were measured using a Seahorse Bioscience XF96 Analyzer (Seahorse Bioscience, North Billerica, MA, USA) as previously described [26].

## SUPPLEMENTARY FIGURES AND TABLES




