# Supplementary material for: Tristetraprolin disables prostate cancer maintenance by impairing proliferation and metabolic function
Source: Oncotarget. 2016 Nov 5;7(50):83462–75. doi: 10.18632/oncotarget.13128 (PMC5341258; doi:10.18632/oncotarget.13128)
Supplement: Supplementary file 1 [file oncotarget-07-83462-s001.pdf]

# Tristetraprolin disables prostate cancer maintenance by impairing proliferation and metabolic function

## SUPPLEMENTARY FIGURES AND TABLES

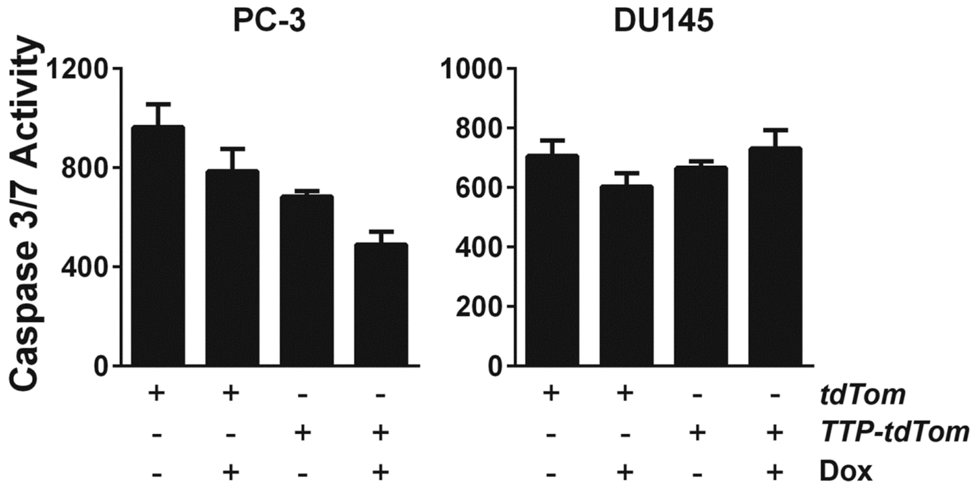


**Supplementary Figure S1: Induction of TTP in prostate cancer cells does not induce apoptosis.** The activity of Caspase 3 and Caspase 7 in PC-3 or DU145 cells expressing *rtTA²* + *tdTom* or *rtTA²* + *TTP* + *tdTom* either untreated or Dox-treated for 48 hr. Caspase activity is measured by luminescence using the Caspase-Glo 3/7 Assay (Promega) (*n*=3). Error bars indicate standard error.

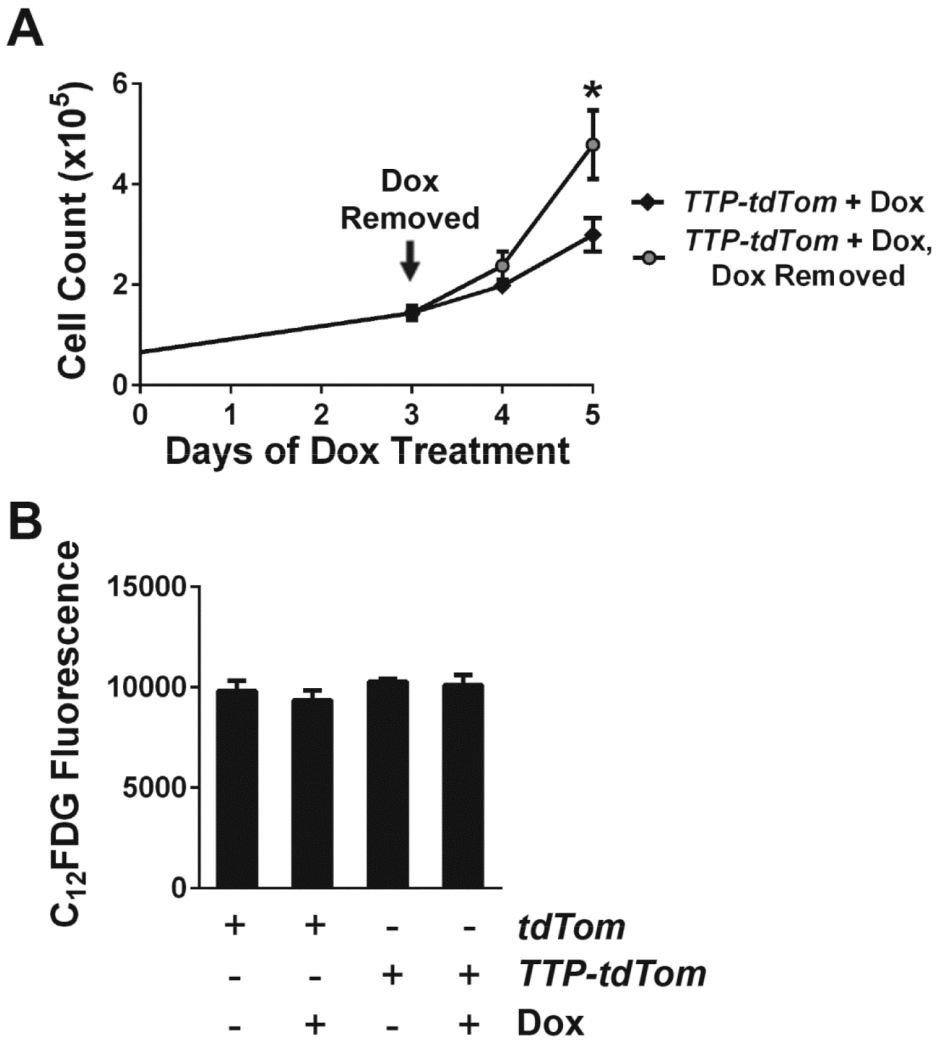


**Supplementary Figure S2: Cell growth impaired by TTP is not caused by senescence. A.** PC-3 expressing *rtTA²* + *TTP* + *tdTom* cells were grown in the presence of Dox for three days. Dox was then removed from half of the wells. The average number of cells for each condition was determined at the indicated time points (*n*=3; *p<0.05, Student's t-test). **B.** Analysis of β-galactosidase activity in PC-3 cells expressing *rtTA²* + *tdTom* or *rtTA²* + *TTP* + *tdTom* +/- Dox treatment for 48 hr. Cells were labeled with 5-dodecanoylaminofluorescein di-β-D-galactopyranoside ($C_{12}$FDG), a fluorogenic substrate for β-galactosidase activity, and analyzed by flow cytometry (*n*=3). Error bars indicate standard error.

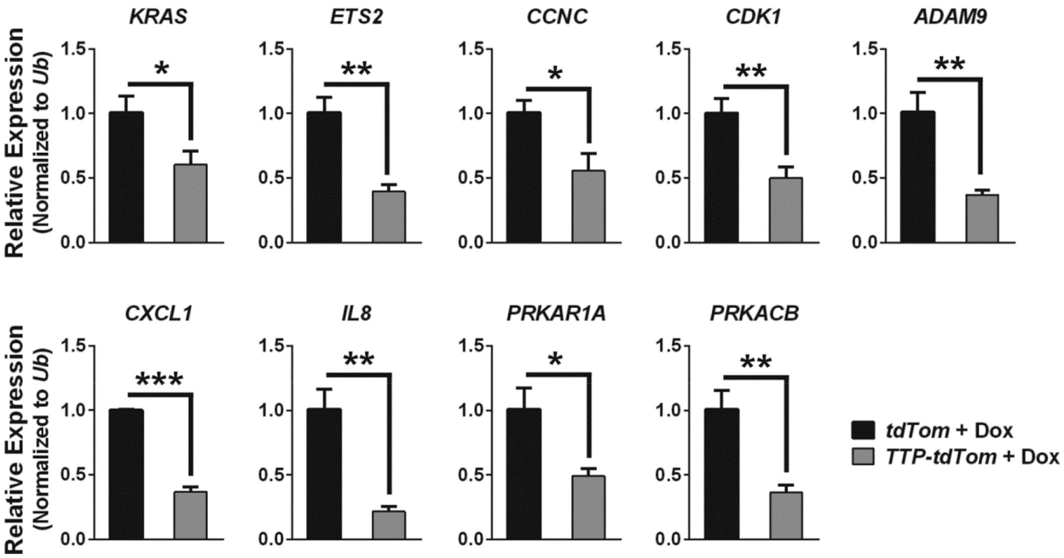


**Supplementary Figure S3: TTP alters the expression of cancer associated genes in PC-3 prostate cancer cells.** qRT-PCR analyses showing the mRNA levels of select cancer-associated genes in Dox-treated (4 hr) PC-3 cells expressing *rtTA*$^2$ + *tdTom* or *rtTA*$^2$ + *TTP* + *tdTom*. qRT-PCR results were normalized to levels of *Ubiquitin* (*Ub*) mRNA. Error bars indicate standard error (*n*=3; *q<0.05, **q<0.01, ***q<0.001, Student's t-test corrected for multiple testing).

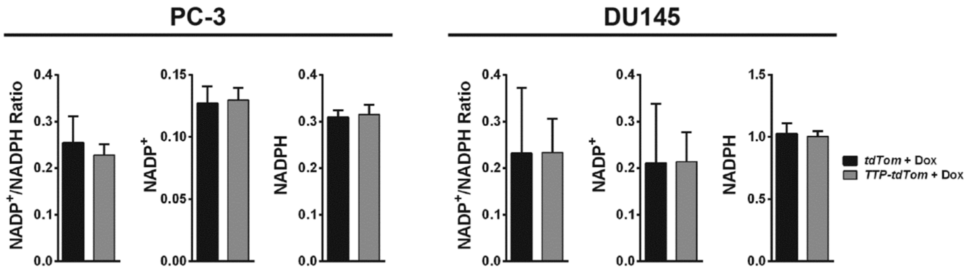


**Supplementary Figure S4: TTP does not affect NADP⁺ or NADPH levels in prostate cancer cells.** Colorimetric assays of NADP⁺ and NADPH levels (pmol/µg protein) in Dox-treated (4 hr) PC-3 or DU145 cells expressing *rtTA²* + *tdTom* or *rtTA²* + *TTP* + *tdTom*. Error bars indicate standard error.

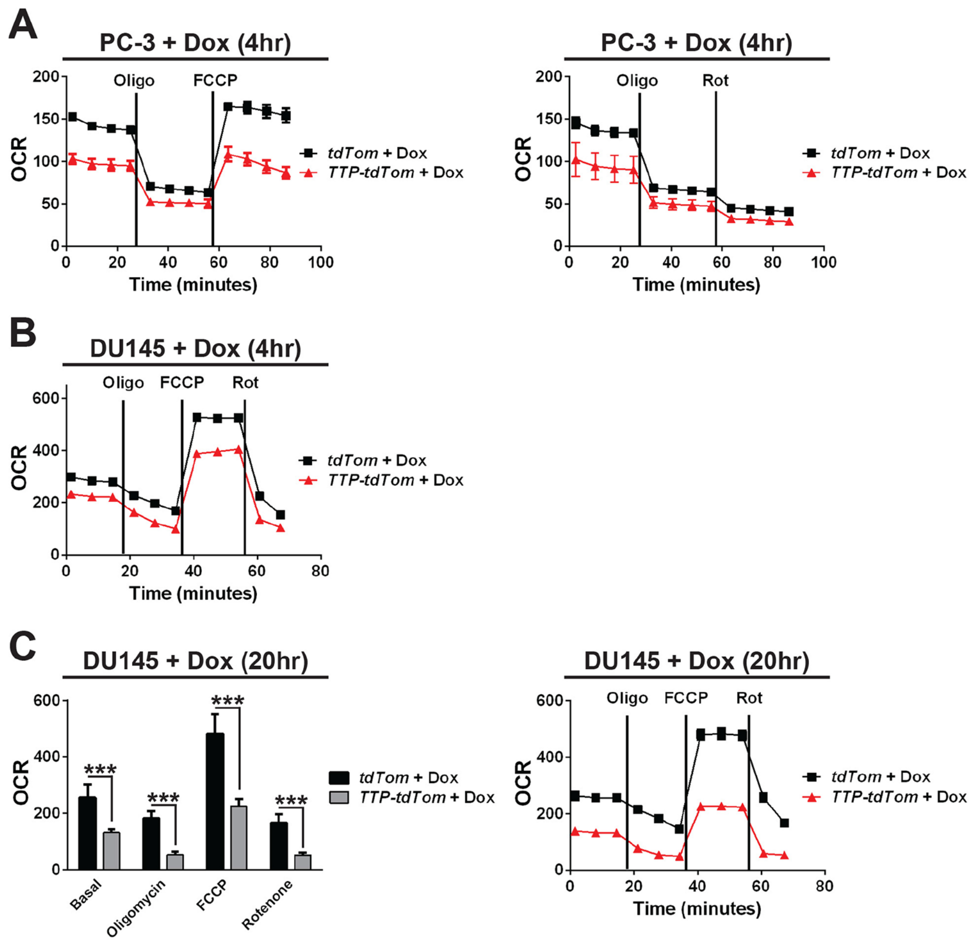


**Supplementary Figure S5: TTP expression impairs OCR in prostate cancer cells. A.** Seahorse OCR (pmol/minute) traces of PC-3 cells expressing *rtTA²* + *tdTom* or *rtTA²* + *TTP* + *tdTom* Dox-treated for 4 hr prior to analysis. **B.** Seahorse OCR (pmol/minute) traces of DU145 cells expressing *rtTA²* + *tdTom* or *rtTA²* + *TTP* + *tdTom* Dox-treated for 4 hr prior to analysis. **C.** Seahorse OCR (pmol/minute) analysis and traces of Dox-treated (20 hr) DU145 cells expressing *rtTA²* + *tdTom* or *rtTA²* + *TTP* + *tdTom*. Basal rates, and rates following the addition of oligomycin (Oligo), FCCP, or rotenone (Rot), are shown ($n \geq 6$; ***p<0.001, Student's t-test). Error bars provided indicate standard error.

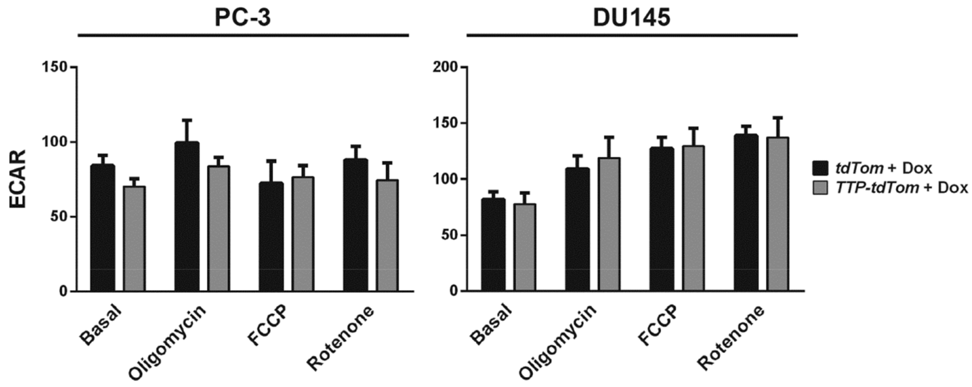


**Supplementary Figure S6: TTP does not alter ECAR in prostate cancer cells.** Seahorse analysis of ECAR (mpH/minute) in Dox-treated (4 hr) PC-3 or DU145 cells expressing *rtTA*$^{2}$ + *tdTom* or *rtTA*$^{2}$ + *TTP* + *tdTom*. Basal rates, and rates following the addition of oligomycin, FCCP, or rotenone, are shown (*n*=6). Error bars provided indicate standard error.

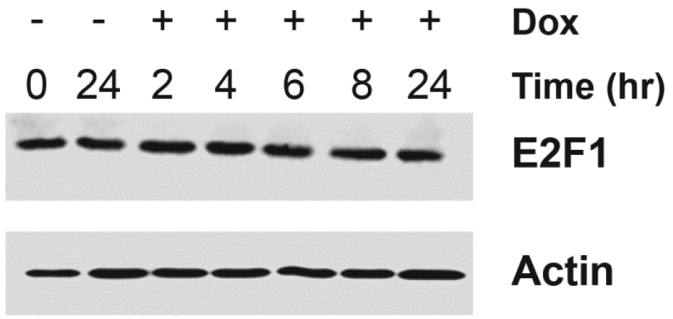


**Supplementary Figure S7: TTP does not affect E2F1 protein expression in PC-3 cells.** Immunoblot blot analyses of E2F1 and Actin levels in PC-3 cells expressing *rtTA² + TTP + tdTom.* Cells were either untreated or Dox-treated for the times indicated.

**Supplementary Table S1: Genes differentially expressed between PC-3 + *rtTA*[2] + *tdTom* and PC-3 + *rtTA*[2] + *TTP-tdTom* cells Dox-treated for 4 hr**

See Supplementary File 1

**Supplementary Table S2: qRT-PCR Oligos**

| Gene | Sequence |
|---|---|
| *ADAM9* | CACGATGATGGGAGAGATTG |
| | TGCACTGCAACTGCTAAA |
| *ADSS* | GGGTAGAGAGTTTGGTGTAAC |
| | AGTGCCAACGCAGTAAAT |
| *BCKDHB* | GTACGGGCAAACTCAGAAA |
| | TCCACCAAAGGCAACATC |
| *CCNC* | GACCTTTGCTCCAGTATGTG |
| | GCAAAGATCCGTTCTGTAGG |
| *CDK1* | GGCCAGAAGTGGAATCTTTA |
| | GAGAGCAAATCCAAGCCA |
| *CMPK1* | ACCTTCAAGGATGGAACAAG |
| | TCATCACTCCTACCACTACTC |
| *CXCL1* | CCCAAGAACATCCAAAGTGT |
| | AAGCTTTCCGCCCATTC |
| *CYCS* | CTTACACAGCCGCCAATAA |
| | AGTCTGCCCTTTCTTCCT |
| *DBT* | CTCAGCACCACTGATCTTAC |
| | GCTACTTCAGGTGGCATTAT |
| *DLAT* | GGCAGTAGAGAAAGGGATTG |
| | GCAGGAGCAACTTTACTAGG |
| *ETS2* | CAGTCTCTCTGCCTCAATAAG |
| | GCACAGCTGCAGGTATAA |
| *GPD2* | TCACAGGGCAGGAATTTG |
| | AGCTGCTGCGTCTTTATC |
| *IDH3A* | TTGTGACCATTCGAGAGAAC |
| | CTCGGTGATGAGCTTGATAC |
| *IL8* | TTGCCAAGGAGTGCTAAAG |
| | CACTCTCAATCACTCTCAGTTC |
| *KRAS* | GCCTTGACGATACAGCTAAT |
| | CCTGCTGTGTCGAGAATATC |
| *PDK1* | AGGCGTCTGTGTGATTTG |
| | TGTGATAGAGATGGGATGGT |
| *PRKACB* | TCAGCAAGGGCTACAATAAG |
| | GAATTGGTTGGTCTGCAAAG |
| *PRKAR1A* | GGAGAGACGGATGTCTATGT |
| | GCTCTCGGTGTTCCATAAAT |
| *TTP* | CAAGACTGAGCTATGTCGGACCTT |
| | CCTGGAGGTAGAACTTGTGACAGA |
| *UB* | ACCTGACCAGCAGCGTCTGATATT |
| | TCGCAGTTGTATTTCTGGGCAAGC |
